# Re-evaluation of the stroke prognostication using age and NIH Stroke Scale index (SPAN-100 index) in IVT patients — the-SPAN 100^65^ index

**DOI:** 10.1186/s12883-018-1126-0

**Published:** 2018-08-29

**Authors:** Cornelia Möbius, Christian Blinzler, Stefan Schwab, Martin Köhrmann, Lorenz Breuer

**Affiliations:** 1 Department of Neurology, Universitätsklinikum Erlangen, Schwabachanlage 6, 91054 Erlangen, Germany; 2 Department of Neurology, Universitätsklinikum Essen, Hufelandstr. 55, 45147 Essen, Germany

**Keywords:** SPAN-100 index, SPAN-100^65^ index, Acute ischemic stroke, I.V.-thrombolysis, Clinical outcome, Symptomatic intracerebral hemorrhage

## Abstract

**Background:**

The SPAN-100 index adds patient age and baseline NIHSS-score and was introduced to predict clinical outcome after acute ischemic stroke (AIS). Even with high NIHSS-scores younger patients cannot reach a SPAN-100-positive status (index ≥100). We aimed to evaluate the SPAN-100 index among a large, contemporary cohort of i.v.-thrombolysed AIS-patients and exclusively among older patients who can at least theoretically achieve SPAN-100-positivity.

**Methods:**

The SPAN-100 index was applied to AIS-patients receiving i.v.-thrombolysis (IVT) in our institution between 01/2006 and 01/2013. Clinical outcome and symptomatic intracerebral hemorrhage rates were compared between SPAN-100-positive and -negative patients. Furthermore we excluded patients < 65 years, without any theoretical chance to achieve SPAN-100-positivity, and re-evaluated the index (SPAN^65^–100 index).

**Results:**

SPAN-100-positive IVT-patients (124/1002) had a 9-fold increased risk for unfavorable outcome compared to SPAN-negative patients (OR 9.39; 95% CI 5.87–15.02; *p* <  0.001). The odds ratio for mortality was 7.48 (95% CI 4.90–11.43; *p* <  0.001). No association was found between SPAN-100-positivity and sICH-incidence (OR 0.88; 95% CI 0.31–2.53; *p* = 0.810).

SPAN^65^–100-positivity (124/741) was associated with an 8-fold increased risk for unfavorable outcome (OR 7.6; 95% CI 4.71–12.22; *p* <  0.001) but not associated with higher sICH-rates (OR 0.86; 95% CI 0.29–2.53; *p* <  0.001).

**Conclusions:**

Also for patients ≥65 years the SPAN-100 index can be a fast, easy method to predict clinical outcome of IVT-patients in everyday practice. However, it should not be used to determine the risk of sICH after IVT. Based on a SPAN-positive status IVT should not be withheld from AIS-patients merely because of feared sICH-complications.

## Background

Age and stroke severity are known major risk factors and predictors for unfavorable outcome in acute ischemic stroke (AIS) patients [1–3]. However, impact of higher age and higher initial National Institutes of Health Stroke Scale (NIHSS)-scores on clinical outcome after AIS and the way how these two factors interact with each other still remain not entirely clear. In particular data showing which individual patient will achieve favorable or unfavorable outcome is sparse. Different scores to predict clinical outcome after AIS have been introduced [4–8]. Most of them are rather complex and may therefore not be extensively used in daily routine. In order to create a fast and practical tool to predict clinical outcome of AIS patients the Stroke Prognostication using Age and NIH Stroke Scale index (SPAN-100 index) was introduced [9]. It simply combines patient age (years) and the NIHSS-Score at stroke onset. Patients with a score ≥ 100 are classified as SPAN-100–positive and those with a score < 100 are designated as SPAN-100-negative. The index was applied to the dataset of the National Institute of Neurological Disorders and Stroke (NINDS)-tPA trials [10] and found to be of good value to predict clinical outcome and risk of intracerebral hemorrhage [9]. Since its introduction the SPAN-100-index has been evaluated several times in different patient cohorts [11–15]. However, previous investigations have in common that they included patients who could not achieve a SPAN-100-positve status at all, e.g. the original SPAN-100-evaluation [9] with a considerable proportion of younger patients. Even with very high NIHSS-scores they were not able to reach a SPAN-Index of ≥100. Including such patients might have increased selectivity of the score potentially leading to an overestimation of its benefit.

The first aim of our study was to re-evaluate reliability of the original SPAN-100 index in an independent, more recent cohort of 1002 i.v.-thrombolysed AIS-patients. The second objective was to exclude younger patients from our patient cohort to exclusively evaluate the SPAN-index among the subgroup of patients who could at least theoretically achieve SPAN-100-positivity.

## Methods

The Erlangen Stroke and Thrombolysis Database is a prospective database of all AIS-patients at the University Hospital Erlangen, Germany. It contains baseline demographic and stroke related data as well as treatment specifics, imaging information and outcome parameters for each stroke patient. Outcome at day 90 was assessed using the mRS (modified Rankin Scale) evaluated by a neurologist as part of the general database independently from the present study using a semi-structured interview either in person or by telephone. Favorable clinical outcome was defined as mRS: 0–2 and/or clinical recovery to the pre-stroke mRS. Unfavorable outcome was defined as a mRS of 4–6. The study was approved by the local ethics committee. 17/1002 patients were lost to follow-up at day 90. Symptomatic intracerebral hemorrhage (sICH) was defined according to the European Cooperative Acute Stroke Study (ECASS) III criteria (sICH-definition 1) [16]. To assure comparability to the derivation cohort we additionally applied the sICH-definition (any documented decline in the neurologic status) used in the original publication [9] (sICH-definition 2). Our institutional guidelines are less restrictive than the European Medicines Agency -licence for tPA, therefore more than half of our patients receive off-label IVT. Besides that all patients were treated and monitored on our stroke unit in accordance with European guidelines [17]. Standard CT-based treatment was performed within the 4.5-h-window. For patients within an extended or unknown time window our institution uses an MRI mismatch-based algorithm as described elsewhere [18]. Follow-up imaging was performed after 24 to 36 h to evaluate for intracerebral hemorrhage and infarct distribution. NIHSS-scores were documented by NIHSS-certified stroke neurologists.

### Study population

For this study we extracted all AIS-patients from the database who consecutively received IVT between 01/2006 and 01/2013. We applied the SPAN-100 index to this cohort to create two groups by adding age (years) to the NIHSS-score on admission. Patients with a score ≥ 100 were classified as SPAN-100-positive, whereas all patients with a score < 100 were designated as SPAN-100-negative. We compared clinical outcomes and sICH-rates of SPAN-100-negative and SPAN-100-positive patients. To identify patients who were eligible for the second part of our study, we subtracted the highest NIHSS-score documented in our patient cohort from the SPAN-100 cut off score of ≥100. Referring to the NIHSS-Coma Scoring Rules (as stated in the original scoring manual developed for the NINDS Trial of tPA for Acute Stroke) this was a NIHSS-score of 35 resulting in a second patient-cohort aged ≥65 years. We again compared clinical outcomes at day 90 and sICH-rates in both groups (SPAN^65^–100-positive and SPAN^65^–100-negative patients).

### Statistical analysis

Statistical analysis was performed using PASW Statistics 19 (SPSS Inc., Chicago, Ill., USA). All data were tested for normality. Categorical variables are presented as frequencies and percentages, whereas continuous data are expressed as mean and standard deviation or as median and interquartile range as appropriate. Intergroup differences were assessed using analysis of variance for normally distributed items, the Kruskal-Wallis test for non-normal data, and the x^2^-test for dichotomous variables. Binary logistic regression was performed with SPAN-100-status as independent variable. *p*-values ≤0.05 were considered significant.

## Results

1002 AIS-patients received IVT at our institution between 01/2006 and 01/2013. After applying the SPAN-100 index 124 (12.4%) were classified as SPAN-100-positive.

### Baseline characteristics

Mean age was 87 years for SPAN-100-positive and 72 years in SPAN-100-negative patients. Median NIHSS-scores on admission were 19 for SPAN-100-positive and 8 for SPAN-100-negative patients respectively. Door-to-needle-time did not differ in both groups. Compared to SPAN-100-negative patients, those in the SPAN-100-positive group were more likely to be women and to suffer from coronary artery disease and atrial fibrillation. SPAN-100-positive patients had worse pre-stroke-mRS scores than SPAN-100-negative patients. Baseline characteristics are summarized in Table 1.

**Table 1:** Baseline characteristics of SPAN-100-positive and SPAN-100-negative patients

Variable	Total (*n* = 1002)	SPAN pos. (*n* = 124)	SPAN neg. (*n* = 878)	*p*-value
Age (years), median (IQR)	73 (18)	87 (8)	72 (17)	< 0.001
Sex (female), n (%)	473 (47.2%)	81 (65.3%)	392 (44.6%)	< 0.001
Risk factors, n (%)				
Hypertension	872 (87.0%)	111 (89.5%)	761 (86.7%)	0.475
Hypercholesterolemia	571 (57.3%)	47 (37.9%)	524 (60.0%)	< 0.001
Nicotine	145 (14.5%)	7 (5.6%)	138 (15.7%)	0.002
Diabetes	330 (32.9%)	40 (32.3%)	290 (33.0%)	0.919
Coronary artery disease	283 (28.2%)	46 (37.1%)	237 (27.0%)	0.025
Previous myocardial infarction	112 (11.2%)	19 (15.3%)	93 (10.6%)	0.128
Previous stroke/TIA	241 (24.1%)	33 (26.6%)	208 (23.7%)	0.501
Atrial fibrillation	412 (41.1%)	86 (69.4%)	326 (37.1%)	< 0.001
door-to-needle time [min.] Median (IQR)	32 (26)	34,5 (24)	32 (26)	0.543
NIHSS-score on admission Median (IQR)	10 (10)	19 (6)	8 (8)	< 0.001
Pre-stroke mRS: 0–1, n (%)	753 (75.1%)	57 (46.0%)	696 (79.3%	< 0.001
Vital signs/laboratory findings				
Temperature on admission [°C] median (IQR)	36.6 (1.0)	36.4 (1.0)	36.7 (0.9)	0.004
Blood glucose on admission [mg/dl] median (IQR)	117 (41)	118 (34)	116 (43)	0.509
Systolic blood pressure on admission [mmHg], median (IQR)	160 (36)	160 (37)	160 (35)	0.748
Diastolic blood pressure on admission [mmHg], median (IQR)	88.5 (23)	85 (19)	89 (23)	0.028
Leucocytes on admission [× 10^3^/μl] median (IQR)	8.40 (3.73)	8.63 (3.61)	8.31 (3.79)	0.073
CRP on admission [mg/l],median (IQR)	4.3 (8.1)	6.85 (13.2)	4.0 (7.8)	0.001
Platelet count on admission [×10^3^] median (IQR)	234 (98)	243.5 (102)	232 (97))	0.205
Triglycerides [mg/dl] median (IQR)	115 (71)	106 (49)	118 (78)	0.055
Stroke subtype				0.001
Large artery disease	92 (9.3%)	8 (6.5%)	84 (9.7%)	
Cardioembolic	440 (44.3%)	88 (71%)	352 (40.5%)	
Small-vessel disease	36 (3.6%)	3 (2.4%)	33 (3.8%)	
Other	36 (3.6%)	0	36 (4.1%)	
Unknown	389 (39.2%)	25 (20.2%)	364 (41.9%)	

*Abbreviations* SPAN = Stroke Prognostication using Age and NIH Stroke Scale, *NIHSS* = NIH Stroke Scale, *TIA* = transient ischemic attack, *CRP* = C-reactive protein

### Outcome comparison of SPAN-100-positive and SPAN-100-negative patients

**Fig. 1:**
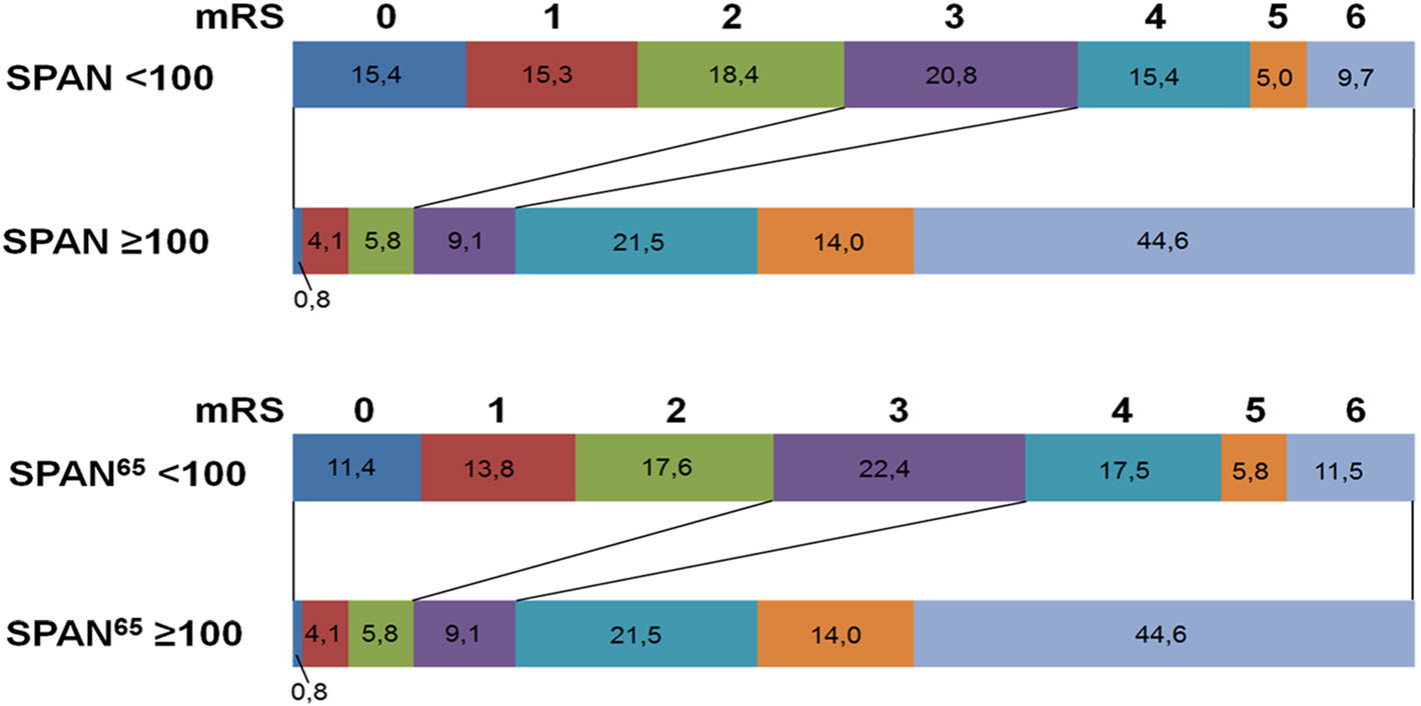
Comparison of functional outcome according to the mRS between SPAN-100-positive and SPAN-100-negative patients as well as between SPAN-100^65^-positive and SPAN-100^65^-negative patients. Abbreviations: SPAN = Stroke Prognostication using Age and NIH Stroke Scale; mRS = modified Rankin scale

SPAN-100-positive patients were more likely to have unfavorable outcomes (80.1% vs. 30.1%; *p* <  0.001), and only 13 of them achieved favorable outcome at day 90 (10.7% vs. 36.9% in the SPAN-100-negative group; *p* <  0.001) (Fig. 1). Mortality during hospital stay (29.8% vs. 6.4%; *p* <  0.001) and after three months (44.6% vs. 9.7%; *p* <  0.001) was significantly higher in SPAN-100-positive patients. Unadjusted binary regression analysis showed a 9-fold increase in the odds of unfavorable outcome for SPAN-100-positive patients and a 7.5-fold increase for mortality after three months (Table 2).

**Table 2:** Association between SPAN-100-positivity and outcome

variable	OR	95% CI	*p*-value
mRS 4–6	9.39	5.869–15.021	< 0.001
mortality at 3 months	7.48	4.901–11.427	< 0.001
sICH-Def. 1	0.88	0.305–2.527	0.810
sICH-Def. 2	1.01	0.389–2.638	0.978

*Abbreviations* SPAN = Stroke Prognostication using Age and NIH Stroke Scale, *mRS* = modified Rankin scale, *sICH* = symptomatic intracranial hemorrhage, *OR* = odds ratio, *CI* = confidence interval

Area under the curve (AUC) for the SPAN-100 concerning mRS 4–6 was 0.74; 95%; *p* <  0.0001; 95% CI (0.71–0.77). Sensitivity and specificity for the cut-off of 100 was 0.27 and 0.96 respectively.

### SICH comparison between SPAN-100-positive and SPAN-100-negative patients

Of 36 sICH (sICH-definition 1) 4 (3.2%) occurred in SPAN-100-positive and 32 (3.7%) in SPAN-100-negative patients, respectively (*p* = 1.000). The binary logistic regression analysis showed that SPAN-100-positive status was not predictive for sICH-incidence regardless if sICH-definition 1 or 2 were applied (Table 2).

### Evaluation of the SPAN-100 index in patients ≥65 years (SPAN-100^65^ index)

741/1002 (74%) patients were ≥ 65 years old. After applying the SPAN^65^–100 index, we identified 617 (83.3%) SPAN^65^–100-negative and 124 (16.7%) SPAN^65^–100-positive patients. Selected baseline characteristics for both subgroups are shown in Table 3.

**Table 3:** Baseline characteristics of SPAN^65^–100-positive and SPAN^65^–100-negative patients

Variable	Total (*n* = 741)	SPAN^65^ pos. (*n* = 124)	SPAN^65^ neg. (*n* = 617)	*p*-value
Age (years), median (IQR)	78 (12)	87 (8)	76 (10)	< 0.001
Sex (female), n (%)	385 (52%)	81 (65.3%)	304 (49.3%)	< 0.001
NIHSS-score on admission,median (IQR)	10 (11)	19 (6)	8 (8)	< 0.001

*Abbreviations* SPAN = Stroke Prognostication using Age and NIH Stroke Scale, *NIHSS* = NIH Stroke Scale, *IQR* inter quartile Range

Outcome results of the SPAN^65^-cohort did not substantially differ from the findings of the total cohort. Mortality during hospital stay (29.8% vs. 7.1%; *p* <  0.001) and after three months (44.6% vs. 11.5%; *p* <  0.001) was higher for SPAN^65^–100-positive patients. Patients in the SPAN^65^–100-positive group were more likely to achieve unfavorable outcomes (80.1% vs. 34.8%; *p* <  0.001) and only 13 patients in the SPAN-100-positive group achieved a favorable outcome at 90 days (10.7% vs. 42,8% in the SPAN 100 negative group; *p* <  0.001) (Fig. 1). SICH (sICH-definition 1) occurred in 4 (3.2%) and 23 (2.7%) of the SPAN^65^–100-positive and SPAN^65^–100-negative patients, respectively (*p* = 1.000). Binary regression analysis revealed patients with SPAN^65^–100-positive status to have an 8-fold increased risk in the odds for unfavorable outcome. AUC for the SPAN^65^–100 was 0.73; 95%; *p* <  0.0001; 95% CI (0.69–0.76). Sensitivity and specificity for the cut-off of 100 was 0.32 and 0.94 respectively. No significant association was found between SPAN^65^–100-status and sICH-incidence regardless which sICH-definition was used. (Table 4).

**Table 4:** Association between SPAN-100^65^-positivity and outcome

variable	OR	95% CI	*p*-value
mRS 4–6	7.58	4.708–12.222	< 0.001
mortality at 3 months	6.18	3.996–3.996	< 0.001
sICH-Def. 1	0.86	0.291–2.526	0.781
sICH-Def. 2	1.22	0.488–3.031	0.674

*Abbreviations* SPAN = Stroke Prognostication using Age and NIH Stroke Scale, *mRS* = modified Rankin scale, *sICH* = symptomatic intracranial hemorrhage, *OR* = odds ratio, *CI* = confidence interval

## Discussion

The SPAN-100 index was introduced by Saposnik et al. to facilitate treatment decisions in stroke patients [9]. With age and stroke severity it combines two main predictors for unfavorable stroke outcome. After applying the index to the patients in the NINDS-tPA trials [10] SPAN-100-positivity was found to be associated with worse outcomes. Besides that higher ICH-rates were seen in SPAN-100-positive compared to SPAN-negative patients whether they had received IVT before or not. An IVT-benefit was described for SPAN-100-negative, but not for SPAN-100-positive patients [9]. However, several conditions may have influenced these results. First, the number of SPAN-positive patients who received IVT was rather small (*n* = 36). Second, the population of the NINDS-trials does not reflect current treatment standards, e.g. patients were randomized to tPA or placebo within 3-h after symptom onset [10].

Several additional studies evaluated the SPAN-100 index after its first introduction [11–15, 19]. Overall they confirmed that SPAN-100-positive patients are less likely to achieve favorable outcome compared to SPAN-negative patients. Only one Chinese study found a low prediction power of the SPAN-100 index for 3- and 12-month outcome [19].

However, data concerning the predictive power of the SPAN-100 index for sICH are inconsistent. Krishnan et al. found higher intracerebral hemorrhage-rates in SPAN-100-positive patients compared to the SPAN-negative patients [9, 13] while Abilleira et al. observed similar sICH-rates [11]. In contrast two other studies comparing performance of different sICH-scores found a poor predictive power of the SPAN-100 index [14, 20].

In our study, we initially re-evaluated the original SPAN-100 index among a larger, more contemporary cohort of 1002 IVT-patients, including off-label treated patients. In terms of outcome our results are in line with previous studies. Patients with SPAN-100-positive status had a 9-fold increased risk of unfavorable outcome at three months.

Irrespective of the used sICH-definition SPAN-100-positive status was not associated with a higher risk of sICH.

Therefore our data do not support the hypothesis by Saposnik et al. who suggested that the SPAN-100 index might help to evaluate the risk of ICH after IVT [9]. This discrepancy might possibly be explained by the fact that advances concerning diagnostic and treatment infrastructure were achieved since the NINDS trials [10]. Continued development of imaging techniques might account for lower sICH-rates despite inclusion of off-label treated patients, e.g. patients with prolonged or unknown time window.

Previous evaluations of the SPAN-100-Index did not differentiate between patients who could a priori reach a SPAN-positive status and those who could not. Even with very high NIHSS-scores, younger patients are unable to reach the SPAN-100 index cut off score of 100. In the analysis by Saposnik et al. 219/624 (35.1%) patients in the SPAN-negative group were younger than 65 years [9]. This might have biased the SPAN-negative group towards a better outcome potentially increasing selectivity of the index and leading to an overestimation of its benefit. In the second part of our study we addressed this issue by removing patients younger than 65 years from our data set and applying the SPAN-100^65^ index to the resulting new patient-cohort aged ≥65 years. Similar to the results of the total cohort, SPAN^65^–100-positive patients had an 8–fold higher risk to reach unfavorable outcome. But even in IVT-patients ≥65 years sICH-rates did not differ between SPAN^65^–100-positive and SPAN^65^–100-negative patients. Hence, unfavorable outcome in our SPAN-100-positive patients was not related to hemorrhagic complications of thrombolytic therapy suggesting that IVT should not be withheld from older stroke patients with high NIHSS-scores merely because of safety reasons. This is in line with results from the SITS-ISTR Registry which show no excess risk of cerebral hemorrhage in patients with NIHSS-score > 25 [21]. Besides that several observational studies indicate safety and efficacy of tPA-treatment in stroke patients > 80 years [22–25] and the results of the third international stroke trial (IST-3) suggest that age should not be a barrier to IVT [26].

Our study has several limitations, primarily the single-center approach and the use of our institutional diagnostic and treatment guidelines as well as the retrospective design. However, including off-label patients might increasingly reflect common daily routine.

## Conclusions

Our findings confirm the value of the SPAN-100 index. Also after excluding younger patients who a priori cannot reach a SPAN-positive status the SPAN-100 index can be an easy to use, readily available calculator in everyday clinical practice to predict clinical outcome in IVT patients. However, in our cohort SPAN-100-positive status was not predictive for the risk of sICH after IVT. Based on a positive SPAN-100 status IVT should not be withheld from patients merely because of feared sICH complications.

## Abbreviations

AIS: Acute ischemic stroke
AUC: Area under the curve
CI: Confidence interval
CRP: C-reactive protein
i.v.: Intravenous
IVT: Intravenous thrombolysis
mRS: Modified Rankin Scale
NIHSS: National Institutes of Health Stroke Scale
NINDS: National Institute of Neurological Disorders and Stroke
OR: Odds ratio
sICH: Symptomatic intracerebral hemorrhage
SPAN-100 index: Stroke Prognostication using Age and NIH Stroke Scale-100 index
SPAN-10065 index: SPAN-100 index evaluated in patients ≥65 years
TIA: Transient ischemic attack
tPA: Tissue type plasminogen activator

## References

[1] Predicting outcome after acute ischemic stroke (2004). An external validation of prognostic models. Neurology.

[2] Konig IR, Ziegler A, Bluhmki E, Hacke W, Bath PM, Sacco RL, Diener HC, Weimar C (2008). Predicting long-term outcome after acute ischemic stroke: a simple index works in patients from controlled clinical trials. Stroke.

[3] Weimar C, Konig IR, Kraywinkel K, Ziegler A, Diener HC (2004). Age and National Institutes of Health stroke scale score within 6 hours after onset are accurate predictors of outcome after cerebral ischemia: development and external validation of prognostic models. Stroke.

[4] Kent DM, Selker HP, Ruthazer R, Bluhmki E, Hacke W (2006). The stroke-thrombolytic predictive instrument: a predictive instrument for intravenous thrombolysis in acute ischemic stroke. Stroke.

[5] Lou M, Safdar A, Mehdiratta M, Kumar S, Schlaug G, Caplan L, Searls D, Selim M (2008). The HAT score: a simple grading scale for predicting hemorrhage after thrombolysis. Neurology.

[6] Ntaios G, Faouzi M, Ferrari J, Lang W, Vemmos K, Michel P (2012). An integer-based score to predict functional outcome in acute ischemic stroke: the ASTRAL score. Neurology.

[7] Saposnik G, Kapral MK, Liu Y, Hall R, O'Donnell M, Raptis S, Tu JV, Mamdani M, Austin PC (2011). IScore: a risk score to predict death early after hospitalization for an acute ischemic stroke. Circulation.

[8] Strbian D, Meretoja A, Ahlhelm FJ, Pitkaniemi J, Lyrer P, Kaste M, Engelter S, Tatlisumak T (2012). Predicting outcome of IV thrombolysis-treated ischemic stroke patients: the DRAGON score. Neurology.

[9] Saposnik G, Guzik AK, Reeves M, Ovbiagele B, Johnston SC (2013). Stroke prognostication using age and NIH stroke scale: SPAN-100. Neurology.

[10] Tissue plasminogen activator for acute ischemic stroke (1995). The National Institute of Neurological Disorders and Stroke rt-PA stroke study group. N Engl J Med.

[11] Abilleira S, Ribera A, Quesada H, Rubiera M, Castellanos M, Vargas M, Gomis M, Krupinski J, Delgado-Mederos R, Gomez-Choco M, et al. Applicability of the SPAN-100 index in a prospective and contemporary cohort of patients treated with intravenous rtPA in Catalonia. Neurologia. 2016;31(9):592–8. 10.1016/j.nrl.2014.10.007 25542499

[12] Almekhlafi MA, Davalos A, Bonafe A, Chapot R, Gralla J, Pereira VM, Goyal M (2014). Impact of age and baseline NIHSS scores on clinical outcomes in the mechanical thrombectomy using solitaire FR in acute ischemic stroke study. AJNR Am J Neuroradiol.

[13] Krishnan P, Saposnik G, Ovbiagele B, Zhang L, Symons S, Aviv R (2015). Contribution and additional impact of imaging to the SPAN-100 score. AJNR Am J Neuroradiol.

[14] Li M, Wang-Qin RQ, Wang YL, Liu LB, Pan YS, Liao XL, Wang YJ, Xu AD (2015). Symptomatic intracerebral hemorrhage after intravenous thrombolysis in chinese patients: comparison of prediction models. J Stroke Cerebrovasc Dis.

[15] Ovbiagele B, Reeves MJ, Nasiri M, Johnston SC, Bath PM, Saposnik G (2014). A simple risk index and thrombolytic treatment response in acute ischemic stroke. JAMA Neurol.

[16] Hacke W, Kaste M, Bluhmki E, Brozman M, Davalos A, Guidetti D, Larrue V, Lees KR, Medeghri Z, Machnig T (2008). Thrombolysis with alteplase 3 to 4.5 hours after acute ischemic stroke. N Engl J Med.

[17] European Stroke Organisation (ESO) Executive Committee and ESO Writing Committee. Guidelines for management of ischaemic stroke and transient ischaemic attack. Cerebrovasc Dis. 2008;25(5):457–507. 10.1159/000131083 18477843

[18] Breuer L, Schellinger PD, Huttner HB, Halwachs R, Engelhorn T, Doerfler A, Kohrmann M (2010). Feasibility and safety of magnetic resonance imaging-based thrombolysis in patients with stroke on awakening: initial single-Centre experience. Int J Stroke.

[19] Pan Y, Jing J, Zhang R, Zhao X, Liu L, Wang H, Liu G, Wang C, Wang Y (2014). Poor performance of stroke prognostication using age and National Institutes of Health stroke Scale-100 to predict 3- and 12-month outcomes of ischemic stroke in China National Stroke Registry. J Stroke Cerebrovasc Dis.

[20] Strbian D, Michel P, Seiffge DJ, Saver JL, Numminen H, Meretoja A, Murao K, Weder B, Forss N, Parkkila AK (2014). Symptomatic intracranial hemorrhage after stroke thrombolysis: comparison of prediction scores. Stroke.

[21] Mazya MV, Lees KR, Collas D, Rand VM, Mikulik R, Toni D, Wahlgren N, Ahmed N (2015). IV thrombolysis in very severe and severe ischemic stroke: results from the SITS-ISTR registry. Neurology.

[22] Mishra NK, Ahmed N, Andersen G, Egido JA, Lindsberg PJ, Ringleb PA, Wahlgren NG, Lees KR (2010). Thrombolysis in very elderly people: controlled comparison of SITS international stroke thrombolysis registry and virtual international stroke trials archive. BMJ.

[23] Ringleb PA, Schwark C, Kohrmann M, Kulkens S, Juttler E, Hacke W, Schellinger PD (2007). Thrombolytic therapy for acute ischaemic stroke in octogenarians: selection by magnetic resonance imaging improves safety but does not improve outcome. J Neurol Neurosurg Psychiatry.

[24] Toni D, Lorenzano S, Agnelli G, Guidetti D, Orlandi G, Semplicini A, Toso V, Caso V, Malferrari G, Fanucchi S (2008). Intravenous thrombolysis with rt-PA in acute ischemic stroke patients aged older than 80 years in Italy. Cerebrovasc Dis.

[25] Vatankhah B, Dittmar MS, Fehm NP, Erban P, Ickenstein GW, Jakob W, Bogdahn U, Horn M (2005). Thrombolysis for stroke in the elderly. J Thromb Thrombolysis.

[26] Sandercock P, Wardlaw JM, Lindley RI, Dennis M, Cohen G, Murray G, Innes K, Venables G, Czlonkowska A, Kobayashi A (2012). The benefits and harms of intravenous thrombolysis with recombinant tissue plasminogen activator within 6 h of acute ischaemic stroke (the third international stroke trial [IST-3]): a randomised controlled trial. Lancet.

